# Nanotracing and cavity-ring down spectroscopy: A new ultrasensitive approach in large molecule drug disposition studies

**DOI:** 10.1371/journal.pone.0205435

**Published:** 2018-10-17

**Authors:** Nicole A. Kratochwil, Stephen R. Dueker, Dieter Muri, Claudia Senn, HyeJin Yoon, Byung-Yong Yu, Gwan-Ho Lee, Feng Dong, Michael B. Otteneder

**Affiliations:** 1 Pharmaceutical Sciences and Therapeutic Modalities, Roche Pharmaceutical Research and Early Development, Roche Innovation Center Basel, Basel, Switzerland; 2 BioCore Ltd, Seoul, South Korea; 3 Korea Institute of Science &Technology, Seoul, South Korea; 4 Picarro, Inc., Santa Clara, California, United States of America; Fred Hutchinson Cancer Research Center, UNITED STATES

## Abstract

New therapeutic biological entities such as bispecific antibodies targeting tissue or specific cell populations form an increasingly important part of the drug development portfolio. However, these biopharmaceutical agents bear the risk of extensive target-mediated drug disposition or atypical pharmacokinetic properties as compared to canonical antibodies. Pharmacokinetics and bio-distribution studies become therefore more and more important during lead optimization. Biologics present, however, greater analytical challenges than small molecule drugs due to the mass and selectivity limitation of mass spectrometry and ligand-binding assay, respectively. Radiocarbon (^14^C) and its detection methods, such as the emerging ^14^C cavity ring down spectroscopy (CRDS), thus can play an important role in the large molecule quantitation where a ^14^C-tag is covalently bound through a stable linker. CRDS has the advantage of a simplified sample preparation and introduction system as compared to accelerator mass spectrometry (AMS) and can be accommodated within an ordinary research laboratory. In this study, we report on the labeling of an anti-IL17 IgG1 model antibody with ^14^C propionate tag and its detection by CRDS using it as nanotracer (2.1 nCi or 77.7 Bq blended with the therapeutic dose) in a pharmacokinetics study in a preclinical species. We compare these data to data generated by AMS in parallel processed samples. The derived concentration time profiles for anti-IL17 by CRDS were in concordance with the ones derived by AMS and γ-counting of an ^125^I-labeled anti-IL17 radiotracer and were well described by a 2-compartment population pharmacokinetic model. In addition, antibody tissue distribution coefficients for anti-IL17 were determined by CRDS, which proved to be a direct and sensitive measurement of the extravascular tissue concentration of the antibody when tissue perfusion was applied. Thus, this proof-of-concept study demonstrates that trace ^14^C-radiolabels and CRDS are an ultrasensitive approach in (pre)clinical pharmacokinetics and bio-distribution studies of new therapeutic entities.

## Introduction

Radioisotopic labeling, especially with radiocarbon, is an excellent tool in pharmaceutical science and has widespread utility in absorption, distribution, metabolism and elimination (ADME) studies in preclinical species and man [1]. There is also a growing list of applications of ^14^C-microdosing and low-level (< 1 μCi) radiotracer/ADME studies to address pharmacokinetics, absolute bioavailability, drug-drug interaction and pharmacodynamics questions for early translational research to man [2, 3, 4]. The familiar, analytical tools are ^11^C positron emission tomography, scintillation counting, and accelerator mass spectrometry (AMS) for carbon-^14^C radiolabeled compounds [5]. AMS provides high sensitivity quantitation of the ^14^C contents in a sample containing any ^14^C-labeled species, often with limited need for internal standards or calibration plots as quantitation is based upon an intrinsic part of the molecule, i.e. the ^14^C label. AMS is arguably one of the most sensitive (and precise) analytical techniques [6].This form of ion beam physics however is still largely similar in operation to the form first described in the late 1970’s with significant improvements in overall size (footprint) [7], and sample processing and introduction systems [8]. None the less, despite the value AMS can bring to clinical (and less often non-clinical) trials, it remains a niche tool that is complex, expensive, and requires skilled facility personnel.

In the search of an alternative to AMS, laser-based spectroscopic methods have been considered since early 1980’s [9]. However the detection sensitivity was initially poor due to the short absorption path-lengths that were available [10]. The availability of the mid-infrared Quantum Cascade Laser (QCL) along with significant improvements in mid-infrared detectors and high reflective mid-infrared coatings on optics have reinvigorated development for the ^14^C Cavity Ring-Down Spectroscopy (CRDS) instrument. In the current application, the technique selects specific molecular ro-vibrational “finger-print” absorptions of radio-carbon dioxide (^14^CO_2_) and its isotopologues (e.g. ^12^CO_2_ and ^13^CO_2_) to quantify the ^14^CO_2_ in the presence of overlapping absorption bands. In brief, the CRDS technique utilizes a high-finesse optical cavity consisting of two or more very high reflective mirrors. Laser light is mode-matched and injected into the cavity and then the laser is shut off starting the “ring-down” event. The intensity of laser light leaking out of the cavity decays exponentially during the ring-down event. When a gaseous sample is introduced between the cavity mirrors, laser light absorbed by the gas will change the characteristic exponential-decay time of the cavity allowing for the quantitation of the gas concentration. When coupled with a sensitive and selective optical measurement model, CRDS achieves sensitivity to ^14^CO_2_ previously only readily accessible by AMS [11]. Several groups have demonstrated systems with sub-contemporary radiocarbon sensitivities; including a study conducted at Lawrence Livermore National Laboratory (LLNL) measuring *in vivo* pharmacokinetics of a ^14^C-labeled xenobiotic in guinea pig [8, 12]. Significantly an Italian group using a different system known as saturated-absorption cavity ring-down (SCAR) achieves AMS sensitivity, but the SCAR technique has a complex light source that require the attention of trained scientists and technicians [13]. Other currently compact but less sensitive CRDS systems are at various development stages for biomedicine and industrial monitoring [14, 15]. CRDS is proving to be part of a “growing revolution” of optical methods for isotope analysis and future improvements in engineer, laser power, spectroscopic models, operational configuration will lead more powerful instruments suited to routine deployment in analytical laboratories [16].

In recent years a growing number of antibodies with novel formats targeting tissue and specific cell populations are entering the drug development portfolio [17]. Bio-distribution studies have become increasingly important during lead optimization and pre-clinical drug development. Biologics such as antibodies, enzymes, antibody drug conjugates, oligonucleotides, and peptides present greater analytical challenges than small molecule drugs due to the mass limitation of mass spectrometry, matrix dependencies of immunoassays and variety of sizes, shapes, and chemical composition. Radiocarbon labeling thus can play an important role in the large molecule quantitation where a quantitative ^14^C-tag is placed on a metabolically stable area of the molecule, assuming there is sufficient detection sensitivity to meet the experimental objectives. In the case of large molecules, this ^14^C-tag can be coupled to a reactive amino acid residue like lysine or cysteine on the surface without having measurable effect on the binding properties or activity. Here, we report the labeling of a model anti-IL17 IgG1 antibody with ^14^C-propionate and the detection of its plasma and tissue time-concentration profiles in mice by CRDS and AMS. To our knowledge, we show for the first time that these ^14^C analytical technologies are equivalent for the detection of trace quantities of a ^14^C-labeled antibody (large molecule) in plasma and tissues as part of bio-distribution studies. In addition, we demonstrate that our results compare well with pharmacokinetic data of the ^125^ iodine labeled anti-IL17 determined previously by liquid γ-scintillation counting [18], a broadly used technique for antibody disposition studies. Furthermore, we highlight the application of tissue perfusion prior to the analytics to determine directly the extravascular tissue concentration of the antibody without the need of correction by the amount of drug in residual blood.

## Materials and methods

### Synthesis of [^14^C] human anti-IL17 IgG1

As test item a human monoclonal antibody which is an IgG1 allotype and is silenced against Fcγ- receptors binding was chosen. The antibody has a high affinity to human IL17 with a K_D_-value of 0.2 nM and 10-fold lower affinity to rodents.

Liquid scintillation counting was accomplished using a HIDEX 300 SL and ULTIMATE GOLD™ cocktail (PerkinElmer Inc., Waltham, MA, USA). Analytical HPLC was performed on an Agilent 1100 series HPLC system (Santa Clara, CA, USA) with UV detection at 280 nm and a coupled RAMONA radioflow monitor (Raytest, Straubenhardt, Germany) for radiodetection. The size exclusion chromatography (SEC) was done with a BioSuite 250, 300 mm x 7.8 mm, 5 μm column by using an eluent of 0.2 M potassium phosphate 0.25 M potassium chloride at a flow rate of 0.5 mL/min. The affinity to the neonatal Fc receptor (FcRn) was checked by FcRn chromatography [19] using a FcRn affinity column with 0.5 ml volume, PN FCRNP-4605 and the following eluents A: 20 mM MES, 140 mM NaCl in Milli-Q water, pH 5.5; and B: 20 mM Tris base, 140 mM NaCl in Milli-Q water, pH 8.8 (gradient: 0–5 min: 80% A, 5–40 min: 80% - 0% A; 40–45 min: 0% A; 45–46 min: 0% - 80% A; 46–51 min: 80%A). The flow rate was 0.5 mL/min. The protein concentration was determined by a UV/Vis BioSpectrometer Basic. The composition of buffer I was 232.5 mM L-Arginine, 119 mM Succinic Acid, 10 mM L-Methionine, 0.06% Tween 20, pH 6.5 and the buffer II was PBS adjusted to pH 8.4 with NaOH. Buffer II (2.5 ml) was added to a solution of anti-IL17 in buffer I (18mg, 400 μL, 0.12 μmol) and filled into a slide-A-lyzer10K dialysis cassettes G2, 10'000 MWCO, 3mL Capacity [Thermo Scientific Prod# 87730]. The solution was dialyzed 5 times against buffer II. A solution of *N*-succinimidyl[1-^14^C]propionate in toluene (Pharmaron, UK, 79.9 kBq (21.6 μCi), 4 μL, 0.36 μmol) was added to an Eppendorf tube (5mL) and the solvent was removed under streaming argon. The brown residue was dissolved in DMSO (20μL), the anti-IL17 solution in buffer II was added, and the reaction solution was shaken for 20 minutes. Then it was backfilled into a hydrated slide-A-lyzer10K dialysis cassettes G2, and dialyzed 5 times against buffer I. 2.4 mL of the ^14^C-labeled mAb were obtained in a concentration of 7.0 mg/mL and a radioactive concentration of 252 kBq/mL (6.8μCi/mL). The quality of the mAb was controlled by SEC and FcRn affinity chromatography.

### Study design and bioanalysis

#### Pharmacokinetic study

All studies were conducted with the approval of the Cantonal veterinary authority of Basel-Stadt in strict adherence to the Swiss federal regulations on animal protection and to the rules of the Association for Assessment and Accreditation of Laboratory Animal Care International (AAALAC). Male adult C57BL/6J mice (Charles River, France) were administered the test compound intravenously (bolus) via tail vein. A dose of 75 nmol/kg containing 2.1 nCi ^14^C -labeled anti-IL17 was administered. Blood was collected into K_2_EDTA coated polypropylene tubes at 1, 3, 7, 24, 48, 72 and 96 hours post dose by venous puncture (sublingual) under deep anesthesia with 5% isoflurane in pure oxygen. Blood was stored on ice and plasma was prepared within 30 min by centrifugation at 15 000 rpm for 5 min at 4°C and frozen immediately. All plasma samples were stored at −20°C.

Organs and blood were collected at terminal time points of 7, 24, 168 and 336 hours after pentobarbital-induced anesthesia (40 mg/kg, i.p.). Organs were collected with and without perfusion. Perfusion was performed transcardially with phosphate buffered saline solution (PBS) with a rate of 8 mL/min for 5 minutes. Thereafter muscle, skin, liver, lung, spleen and kidneys were removed immediately, rinsed with PBS, snap frozen and stored at −20°C.

#### Cavity ring down spectroscopy

A detailed description of the Wavelength Scanned Cavity Ring-Down Spectroscopy (WS-CRDS) technology can be found somewhere else [12, 20]. A brief introduction to the newly developed commercial WS-CRDS instrument for radio-carbon ^14^C measurements by Picarro, Inc. Santa Clara, California, USA (currently in alpha testing) is provided in the following section. Briefly, laser light is absorbed by molecules at specific wavelengths, and the absorption strength is proportional to the light-molecule interacting path-length. In a typical CRDS system, the light can be bounced back-and-forth thousands of times between multiple high-reflective mirrors. Hence the equivalent path-length can be longer than 10 km, which is essential to boost the absorption intensity up to the detectable range. The technology used here is WS-CRDS which has been developed by Picarro Inc. Santa Clara, California, USA and proven for the trace detection of small molecules, like rare isotopes of H_2_^17^O and site-specific isotopomers of ^15^N^14^NO and ^14^N^15^NO [21].

**Fig 1:**
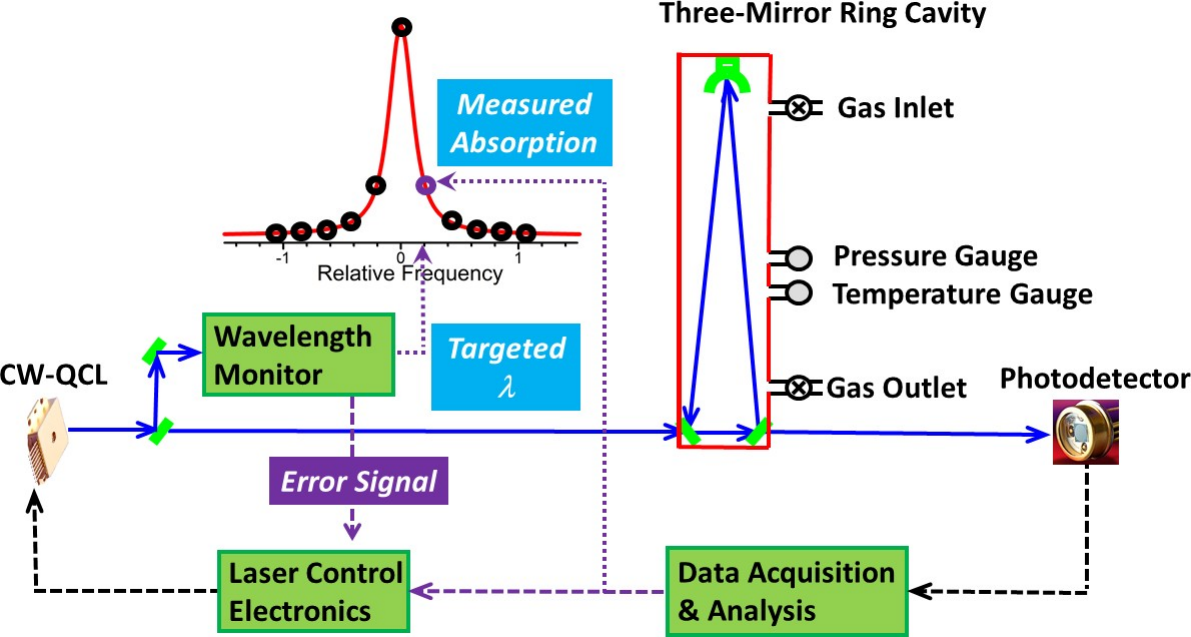
Schematic diagram of the Wavelength-scanned Cavity Ring-Down Spectroscopy(WS-CRDS).

Fig 1 shows a schematic diagram of the principles of the Wavelength-scanned Cavity Ring-Down Spectroscopy (WS-CRDS) in general. The key components are: a highly reliable continuous-wave (CW) laser (DFB QCL) that can be tuned accurately and repeatedly over a spectral range of interest, a wavelength meter that can control the targeting of the laser wavelength, and a precisely aligned optical cavity with three reflective mirrors (>99.99%) [20]. The sequence of analysis events are as follows: first the wavelength meter sends the request to the laser control unit to tune the laser to a specific wavelength that is resonant to the optical cavity. Next, the light is injected into the cavity. After the light signal received at the photodetector reaches a predefined threshold, the control unit will turn off the laser. The “ring-down” time is then measured by recording the light intensity leaked out of the cavity as it decays exponentially over time by the light transmitted through one of the mirrors (optical loss). The optical loss is calculated using the characteristic ring-down time. By scanning the wavelength of the laser over a spectral feature of interest and measuring the ring-down time and thus the optical loss at each wavelength, the detailed profile, area and height of an individual absorption line can be produced, from which the concentrations of each isotopologue (^14^CO_2_, ^13^CO_2_ and ^12^CO_2_) is determined [12].

For the sample preparation for CRDS analysis, a 40 μL aliquot of plasma (1:10 or 1:100 dilution of original mouse plasma diluted with human heparin plasma) was first transferred to a 8 mm x 5 mm tin capsule (Costech, Valencia CA) and then dried in a convection oven held at 60°C for about 1.5 hrs. Similarly, an 80 μL of tissue homogenate (4 volumes water to wet mass mechanically dispersed with a disposable shearing blade) was prepared by the same technique except that samples were placed in larger tin capsules (9 mm x 10 mm) to ensure the sample did not escape from the capsule during drying. To obtain a representative sample, the disposal tip of a 200 μL pipette were widened by cutting off the final few millimeters of the tip. Some skin samples, due to difficulty in homogenization, were analyzed after drying and dissecting as a 4–6 mg piece of the tissue strip. The tin capsules were closed (compressed) with tweezers and loaded onto a 50-place autosampler that feeds directly into the oxidizer tube (Costech, but repackaged by Picarro). In order to make a successful measurement, a minimum of ~1.2 mg carbon dioxide is required from each sample for the current setup. This requirement is proportional to the cavity size and the preselected gas pressure for the cavity. The auto-sampler is remotely controlled by the computer to drop one tin-cup at a time into the combustion module and then flash combustion occurs, converting organic carbon into gaseous CO_2_ streamed with the high purity helium (99.9999%). CO_2_ is then cryogenically trapped in a copper coil submersed in liquid nitrogen Dewar (connected to a 160 L L2L cylinder that was self-leveling) and non-condensable gases are removed under vacuum after the initial trapping. The carbon dioxide is then thermally released into the optical cavity for the ring-down measurements. With a very conservative set-up for the first real time pharmacokinetic studies of CRDS, the measurement time for each sample was set ~20 min each, or a throughput of ~50 samples per day. We expect we can increase the throughput with optimization of system conditions (e.g. volume of the system plumbing, pump-down time for trapped CO_2_, conditions for the thermal release of the trapped carbon dioxide, a dynamic ring-down acquisition time and other parameters). The measured ^14^C (in units of Modern, which is the ratio of ^14^C/^12^C) is directly reported from the concentration of ^14^CO_2_/^12^CO_2_. A set of plasma matrix standards was run early in the instrument installation. The slope (0.959) and intercept (-0.490) generated during this run was applied to all future data. The quality of the results were followed with standards placed in the carousel to serve as Quality Controls (QCs) (we did not operate with formal acceptance criteria). The efficiencies for flash combustion, trapping and thermal release are all close to 100% (efficiency was evaluated from analysis of exact mass standards at Picarro) which enables the determination of total carbon content from the samples. Recovery of the drug component is assumed to be 100% since there is no processing other than water removal.

Samples were analyzed in batches up to 50 samples, the capacity of the autosampler on this instrument (larger ones are commercially available). With most sample sets, accepted standards (Oxalic acid II, NIST) were included. In addition, secondary standards of ^14^C enriched oxalic acids (dubbed Ox12 and Ox100) were analyzed as QC’s for accuracy. These standards were obtained as a gift from Lawrence Livermore National Laboratory (LLNL, Drs. Ognibene and Buchholz). The cumulative probability distribution showed these standards to have Modern values of 12.3829 and 102.0357 for Ox12 and Ox100, respectively (personal communication with Ted Ognibene, LLNL). Additionally, a set of human plasma standards (BioChemmed, Winchester Virginia, USA) spiked with a radiolabeled research pharmaceutical (identification not provided here) was prepared at BioCore that ranged from contemporary levels of ^14^C (~1 Modern; no added ^14^C-drug) to 16,658.714 Modern, corresponding to 0.516 to 9,035.687 dpm/mL of plasma. The highest standard was measured by liquid scintillation counting with lower concentration predicted by quantitative serial dilution with blank (no added ^14^C) plasma.

#### Accelerator mass spectrometry

Plasma and tissue samples were converted to elemental carbon (graphite) using well proven two-step oxidization and reduction chemistry carried out in disposal quartz tubes (combustion) and reaction vials (reduction) to avoid cross-contamination [22, 23]. The plasma was analyzed after a 1:100 dilution with human heparin plasma (BioChemmed, Winchester Virginia, USA) to extend the volume and to ensure the samples did not exceed the tested dynamic range of the AMS instrument. After reduction of the carbon dioxide generated from the sample, the resulting graphite that formed on the surface of an iron powder was mixed using a metal rod with stirring, and then pressed into an aluminum target holder that was placed inside a cathode housing. Measurements were performed on a 6 MeV High Voltage Engineering Europa Instrument (HVEE Tandetron ME AMS System; Amersfoort, the Netherlands). For the normalization of the AMS measurements, 5% of total number of samples were prepared from SRM4990C (Oxalic acid II) obtained from the National Institute of Standards and Technology (NIST) (Gaithersburg, Maryland, USA). Secondary standards of C3 and C8 standards obtained from the International Atomic Energy Agency (IAEA; Vienna Austria) were evaluated with the batch to assess any irregularities in the pre-treatment process. The ratio of measured ^14^C/^12^C was corrected using identically prepared Oxalic acid II (Oxalic acid, NIST SRM4990C) measurements and the raw data converted to Modern carbon value using KIST CAL 1.0 internally designed software. The AMS measurement times were set to collect at a minimum, 10,000 “counts” (^14^C atoms) which, because of Poisson statistics, is equivalent to 1% counting precision [6].

### Data analysis

The derived Modern values from AMS or CRDS measurements were converted into dpm/mL and nM units. The accepted definition of 1.0 Modern is 13.56 dpm/g Carbon or 6.11 fCi/mg Carbon. Mouse plasma was diluted 100-fold with human heparin plasma. The human plasma was determined to possess 42 mg Carbon/mL by elemental analysis.

For plasma samples, the following Eqs (1 and 2) were used. Mod is a shortened version of the Modern unit ratio.

$$plasma\;concentration\;\left[\frac{dpm}{mL}\right]=Dil.Factor*Mod*42\frac{mg\;C}{mL}*13.56\frac{dpm}{g\;C}*0.001$$ (1)

$$antibody\;plasma\;concentration\;[nM]=\frac{Dil.Factor*Mod*42\frac{mg\;C}{mL}6.11\frac{fCi}{mg\;C}*0.001}{specific\;activity\;of\;antibody\;(\frac{nCi}{nmol})}$$ (2)

The tissue samples were analyzed without any additional carbon dilution. The natural background was set at “zero” drug concentration (1.0 M applied in the current case with this value confirmed by parallel AMS analysis and consistent with global ^14^C levels) and subtracted from the concentration calculation. For the tissue samples, the following Eq (3) was applied.

$$antibody\;tissue\;concentration\;[nM]=\frac{Dil.Factor*Mod*tissue\frac{mg\;C}{mL}6.11\frac{fCi}{mg\;C}*0.001}{specific\;activity\;of\;antibody\;(\frac{nCi}{nmol})}$$ (3)

The tissue carbon concentrations were derived by taking the tissue volumes from the mouse [24] with the following mean measured carbon percentages based upon human estimates [14], e.g. kidney (0.525 mL,12.9%), liver (1.93 mL, 14.4%), lung (0.204 mL, 10%), muscle (11.3 mL, 10.7%), skin (5.02 mL, 22.7%), spleen (0.127 mL, 11.1%). Tissue ratios (antibody bio-distribution coefficients ABCs) were calculated by dividing the tissue concentration by the corresponding plasma concentrations. The mean tissue ratios are means across time. The ABCs corrected for residual plasma volumes in tissue were derived by dividing the extravascular tissue concentrations [25] by the plasma concentration. The extravascular tissue concentrations of the antibody was calculated by the following equation.

$$extravascular\;antibody\;tissue\;concentration=\frac{C_{tissue}xV_{tissue}-C_{plasma}xV_{residual\;plasma}}{V_{tissue}-V_{residual\;plasma}}$$ (4)

where C_tissue_, V_residual plasma_, V_tissue_ represent tissue concentrations, residual plasma volume in the corresponding tissue and tissue volumes, respectively. The applied residual plasma volumes in tissue in this study were either reported by Eigenmann et al [11] or Boswell et al [26]. The used tissue volumes were published by Eigenmann et al [11].

#### PK modeling

A linear two-compartment model was used to characterize the time-course of the plasma anti-IL17 antibody. The drug is directly administered into the central compartment with IV dosing and undergoes linear distribution to a non-specific peripheral compartment and linear elimination from the central compartment. The following differential equations were used to describe the pharmacokinetics.

$$\frac{{dA}_{c}}{dt}=-CL*C_{c}-Q*(C_{c}-C_{p})$$ (5)

$$\frac{{dA}_{p}}{dt}=Q*(C_{c}-C_{p})$$ (6)

$$C_{c}=\frac{A_{c}}{V_{c}};\;C_{p}=\frac{A_{p}}{V_{p}}$$ (7)

A_c_ and A_p_ are the amounts of the drug in the central and peripheral compartments. The central and peripheral volumes are V_c_ and V_p_ and the plasma clearance and inter-compartmental clearance are CL and Q. To obtain anti-IL17 mAb concentration in molar units a molecular weight of 150 000 g mol^-1^ was applied and a specific activity of 1.24 nCi nmol^-1^ for the blended dose. The analysis was performed using the Phoenix® computer program (Phoenix® 6.4.0.768, Certara L.P, Cary, North Carolina) and the maximum likelihood estimation method First-Order Conditional Estimation Extended Least Squares (FOCE-ELS). The inter-subject variability was implemented in V_p_. No other inter-subject variability could be successfully implemented due to the composite pharmacokinetic profiles. The inter-individual variation in V_p_ was modeled as exponential error model as follows:

$$V_{pi}=V_{p}*e^{\eta_{vpi}}$$ (8)

where V_pi_ is the individual central volume of subject i, i = 1, …,n (n = total number of subjects), is the mean population V_p_, and η_*vpi*_ is the random variable with mean zero and variance of ω^2^ (V_p_). For residual variation, an additive and multiplicative error model was considered. The multiplicative error was used in the final model:

$${Cobs}_{ij}={Cpred}_{ij}*(1+\varepsilon_{ij})$$ (9)

where Cobs_ij_ is the observed concentration in individual i at time j, Cpred_ij_ is the individually predicted concentration, and ε the random variable with mean zero and variance of σ^2^. For assessment of the goodness of fit, the precision of the parameters estimates, minimum objective function value (OFV) (-2 times the log likelihood function), Akaike information criterion, examination of residuals and visual inspection of the fitted curves were used. To assess the model predictive performance, prediction corrected visual predictive checks were performed.

## Results and discussion

### Radiosynthesis

**Fig 2:**
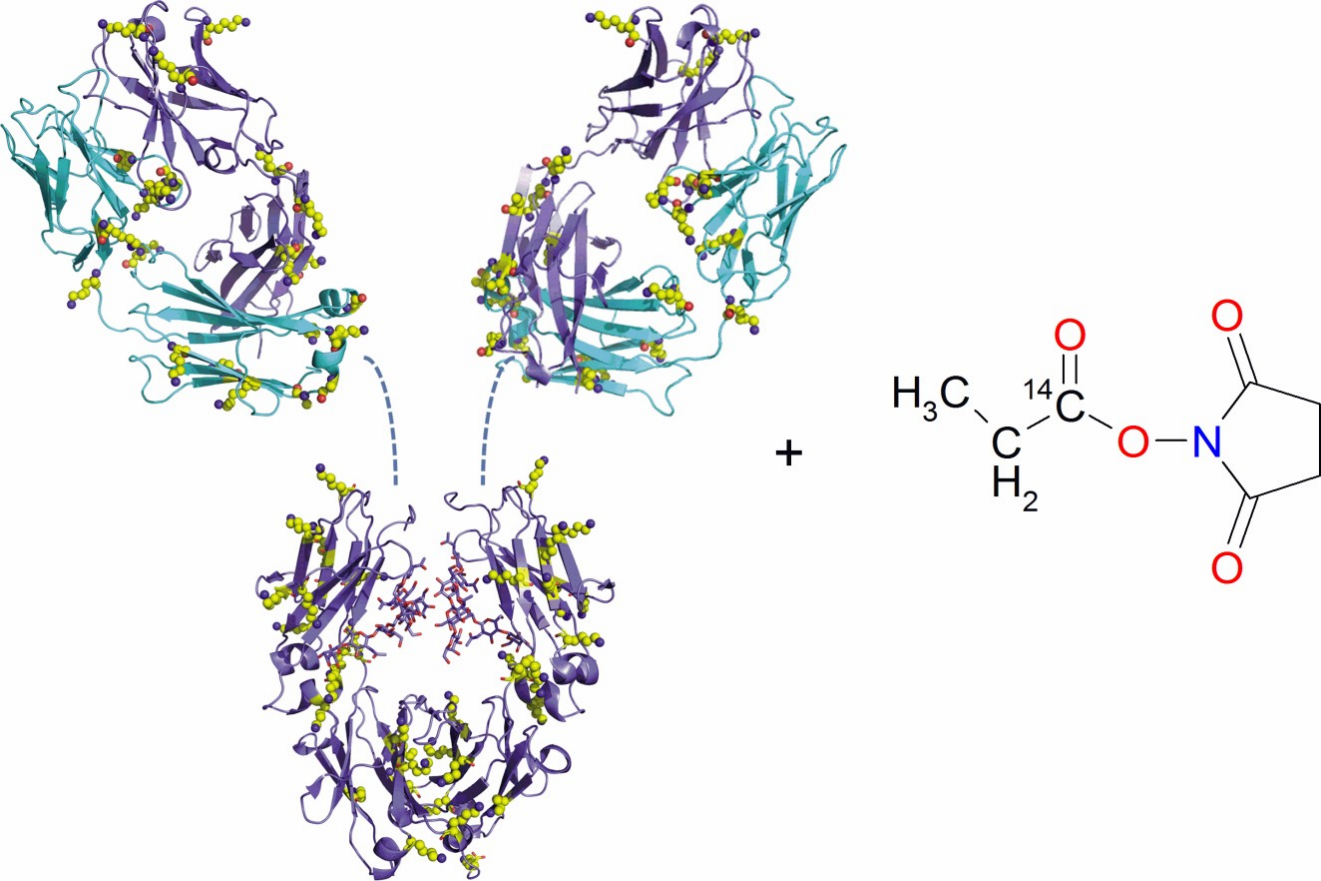
Schematic view of the anti-IL17 antibody and chemical structure of the labeling reagent *N*-succinimidyl [1-^14^C]propionate ([^14^C]NSP). The lysine side chains of the anti-IL17 antibody are shown as spheres colored in yellow, which could carry the ^14^C-propionate tag after the labeling reaction with [^14^C]NSP. The light and heavy chains of the anti-ILl7 are shown in light blue and violet, respectively. The cartoon representation of the Fab is based on coordinates of an in-house X-ray structure. For the Fc the coordinates of the pdb entry 1HZH were used. The hinge region is shown as dashed lines. The image has been generated using the program Pymol (The PyMOL Molecular Graphics System, Version 2.0 Schrödinger, LLC).

Anti-IL17 antibody was labeled with *N*-succinimidyl [1-^14^C]propionate ([^14^C]NSP). Fig 2 presents the schematic view of the anti- IL17 antibody and the chemical structure of the labeling reagent [^14^C]NSP.

The lysine side chains of the anti-IL17 antibody, which could be tagged by [^14^C]NSP, are shown as spheres colored in yellow. As shown in Fig 2, the light (light blue) and heavy (violet) chains of the anti-IL17 contain 13 and 31 lysines, respectively. Besides the fact that the labeling reagent [^14^C]NSP is available from commercial suppliers it comprises several advantages. Propionate is a very small tag, especially compared to other labeling reagents such as fluorescence tags or metal chelators and therefore reduces the probability of altering the structure, activity and overall properties of the antibody. The lysine residues are better accessible with such a small and sterically unhindered functional group and allows for a fine tuning of the labeling reaction. For the ^14^C labeling of anti-IL17, 3 equivalents of [^14^C]NSP were dissolved in small amounts of DMSO. Then a pH 8.4 PBS solution of the anti-IL17 mAB was added and shaken for 20 min before the solution was dialyzed to the pH 6 formulation buffer. A pH 8.4 buffer was selected for the labeling reaction to increase the reaction rate while keeping the hydrolysis of the activated ester low. The specific activity was determined to be 35.9 kBq/mg (0.97 μCi/mg) and the molar activity 5.40 GBq/mmol (146 mCi/mmol) which corresponds to 2.5 labels per antibody. Ten equivalents of the [^14^C]NSP resulted in a labeling degree of 8 ^14^C-propionates per mAB demonstrating that the labeling of the antibody can be fine-tuned with this method. Quality control by size exclusion chromatography revealed a purity of the labeled antibody of 98.3% by UV at 280 nm with 1.7% aggregates, very similar to the native anti-IL17 antibody which was employed for the reaction. The affinity to the neonatal Fc receptor was also tested which is indicative for the PK behaviour of the mAB. The native mAB and the labeled mAB exhibited a very similar retention time on the FcRn affinity column (difference <0.1 min) demonstrating that the binding properties of the antibody did not change during the labeling procedure.

### Bioanalysis by CRDS and AMS

**Fig 3:**
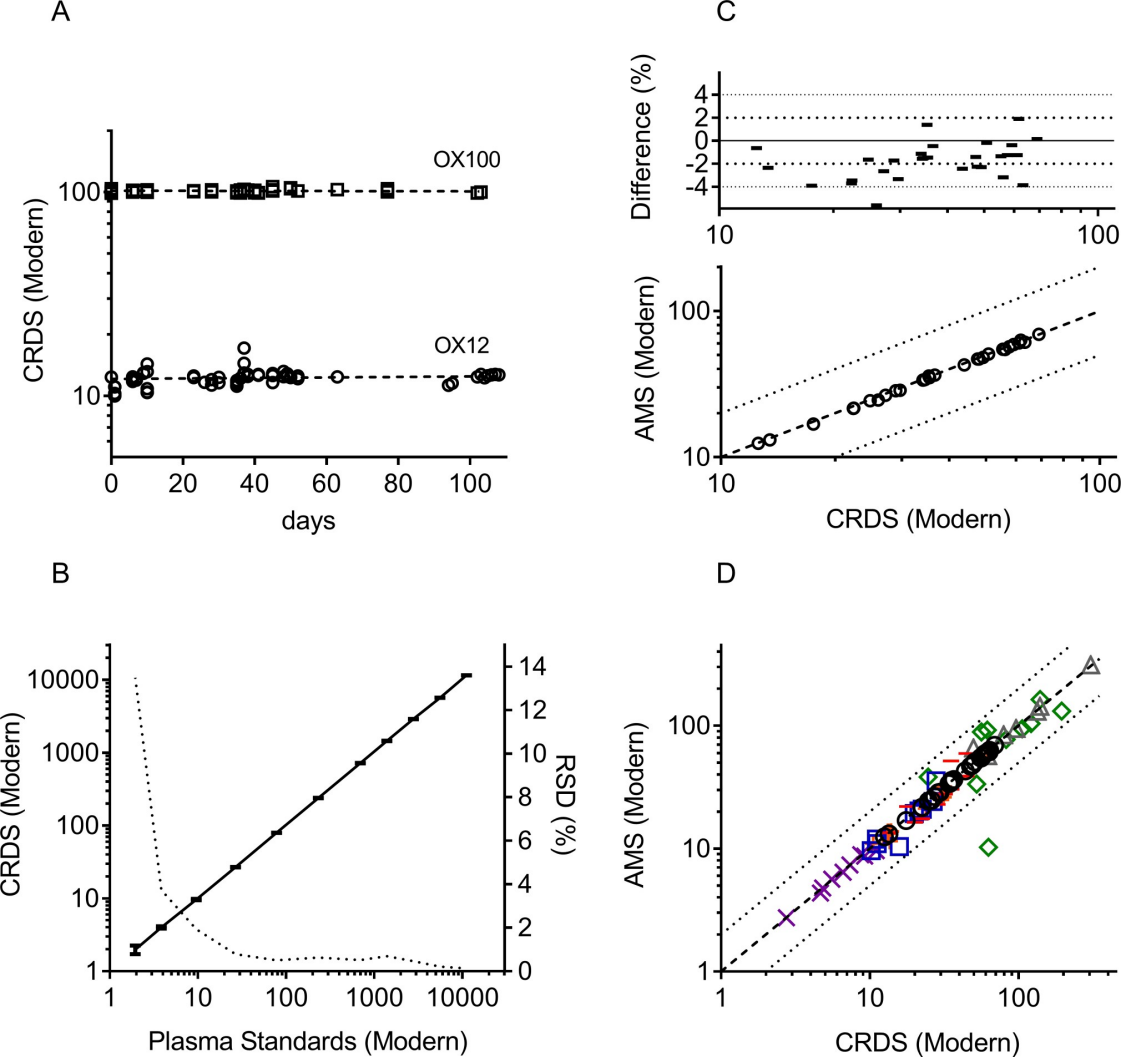
Performance of CRDS technology and correlation between CRDS and AMS measurements. A. Stability of the CRDS determination using Ox12 and Ox100 secondary standards over several months (dotted lines represent linear regression lines) B. CRDS linearity and relative standard deviation (dotted line) for a standard concentration series (small line symbols are standard deviations of 5 measurements per standard and black line represent the regression line) in human heparin plasma ranging from 1.952 to 11,106.81 Modern (0.0516 dpm/mL to 6,023.791 dpm/mL). C. Difference between AMS and CRDS spectrometry for plasma (1:100 diluted, Modern) samples of anti-IL17 derived by using whole body perfusion in mice (lines in the upper panel indicate a change of 2%). D. Correlation between AMS and CRDS spectrometry for plasma and tissue (Modern) for anti-IL17 derived by using whole body perfusion in mice. Plasma (black circles), kidney (orange cross), liver (violet x), spleen (read line), lung (green diamond), muscle (blue square) and skin (grey triangle). In C and D (right panels), the dashed line represents the line of unity and the dotted lines are a 2-fold error margin.

The concentrations of anti-IL17 in plasma and tissue were determined by CRDS after intravenous administration to male adult C57BL/6J mice. The dose was 75 nmol/kg anti-IL17 containing 2.1 nCi ^14^C -labeled anti-IL17 as a tracer. The time points for blood sampling were 0.04, 0.13, 0.29, 1, 2, 4, 7 and 14 days post dose and for lung, skin, liver, spleen, kidney, muscle tissues 0.29, 1, 7 and 14 days, respectively. In addition, whole body perfusion was performed prior to the tissue sampling. For comparison to the CRDS data, AMS measurements were conducted for plasma and tissue samples from the perfused animal group. Fig 3 shows CRDS performance data and the correlation between CRDS and AMS.

In Fig 3A, CRDS accuracy and stability over time are given using ^14^C enriched Oxalic Acid standards. The standards were described by the following regression plots, 0.003474 ± 0.0033 (standard errors SE) and 0.007287 ± 0.0105 (SE) for the slopes and 12.11 ± 0.209 (SE) Modern and 101.6 ± 0.529 (SE) Modern for the intercepts, for Ox12 and Ox100, respectively. This demonstrates that the CRDS measurements are very robust over several months. In Fig 3B, the CRDS linearity across a concentration range from 1.952 to 11,106.81 Modern (0.0516 dpm/mL to 6,023.791 dpm/mL) is presented using standards prepared in human heparin plasma. The top end of the measurement capability is higher but these measurements were not required for this study. The slope of the linear regression for the plasma matrix standard was 1.032 ± 0.00285 with a 1:5700 dynamic range. The correlation factor r^2^ was 0.9999. The relative standard deviation was < 1% at levels above 25 Modern increasing to 15% at 1.952 Modern as determined from 5 separate runs. This plot accounts for all the sources of error in the system, including pipetting, drying, combustion and the laser cell. These data show the CRDS method to be linear and precise. Fig 3C and 3D give the correlation between CRDS and AMS spectrometry, a well-accepted technology for ^14^C-measurements, for the biological samples. Fig 3C presents the difference between CRDS and AMS measurements for 1:100 diluted plasma samples in a parallel analysis. After 100-fold dilution, Modern values ranged from 12 to 69 Modern, values which were well within the dynamic range of both instruments. For plasma (black circles in Fig 3C and Fig 3D), a strong correlation could be observed with a correlation factor r^2^ of 0.9989. The discrepancy (Fig 3C) between the two instruments exceeded 5% difference for 1 of 29 data points (N = 29). The mean average across these plasma samples for the CRDS was 1.8% higher than the equivalent measurement by AMS. A mean difference of 1.8% shows equivalency of the detection platforms despite very different sample processing procedures.The correlation for all tissues is given in Fig 3D for the study samples of the perfused animal group. Greater variability was seen for the tissue samples (Fig 3D) with correlation factors of 0.9808, 0.9731, 0.5951, 0.8450, 0.9935 and 0.7224 for kidney, liver, lung, muscle, skin and spleen, respectively (Fig 3D). The greater variability is presumably due to difficulties in preparing a homogeneous sample for the measurement. The 2.1 nCi dose for the ^14^C-labeled anti-IL17 was based upon prior knowledge about its pharmacokinetics and bio-distribution [11]. As it turned out, the CRDS component of the study could have been completed with a high pCi-sized dose as the found lower limit of quantification LLOQ was 2.013 Modern (including background of 1.041 Modern) or 0.570 dpm/mL (above background). For the LLOQ determination, we examined 45 blank plasma samples and found them to report a mean Modern of 1.041 with a SD of 0.162. The limit of quantification was set as a multiple of 6 times the SD [6].

### Pharmacokinetics

**Fig 4:**
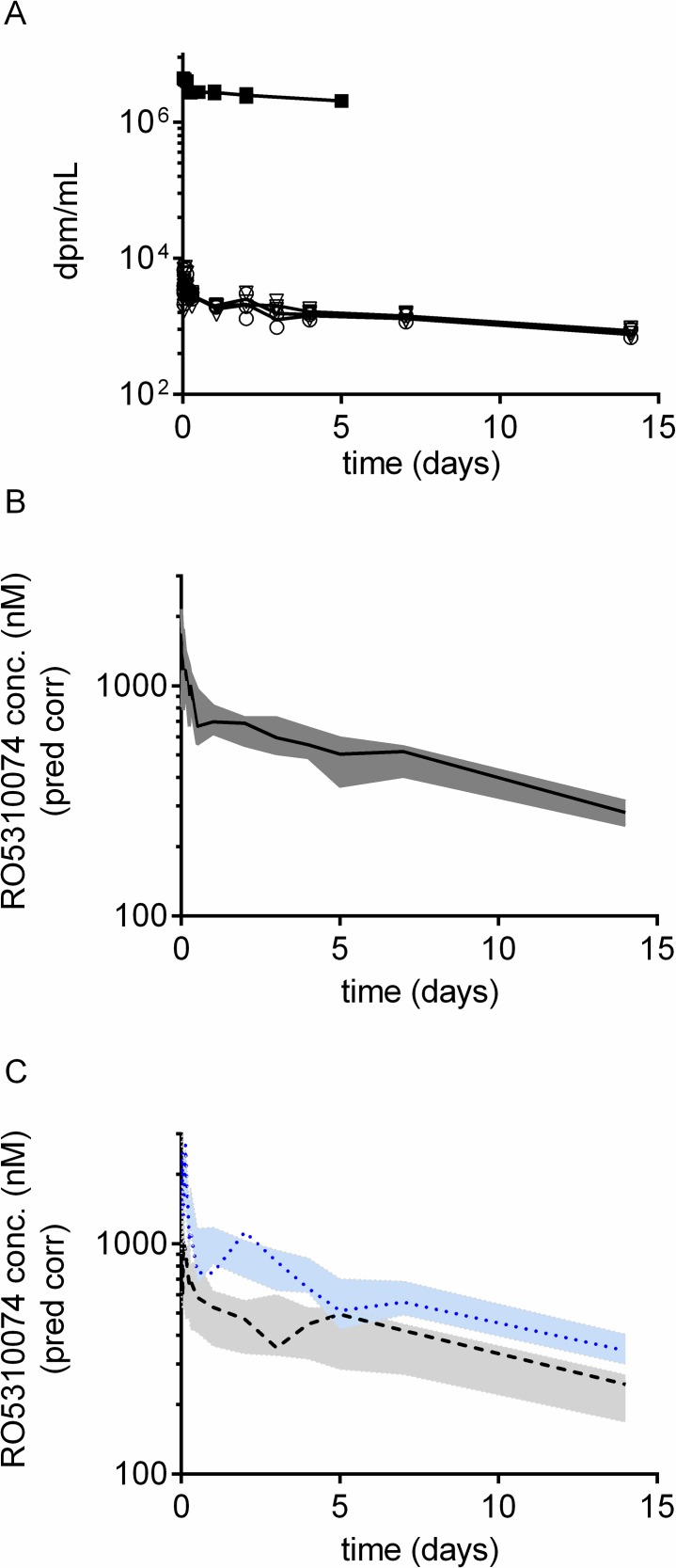
Observed and predicted plasma concentration time profiles in mice after IV dosing of 75 nmol/kg containing 2.1 nCi ^14^C -labeled anti-IL17 as nanotracer. A. Radioactivity levels (dpm mL^-1^) in mouse plasma determined by CRDS (triangles) and AMS (circles). For comparison, the radioactivity levels (A) of anti-IL17 in mouse plasma are given after a IV dose of 66 nmol/kg anti-IL17 containing 600 nCi ^125^I-labeled anti-IL17 (squares), for which the experimental details where reported elsewhere [11]. B and C Prediction corrected visual predictive check for the plasma concentration-time profiles of the anti-IL17 (all data). B. The solid black line represents the median prediction-corrected plasma concentration and the dark grey area represents a simulation-based 95% confidence interval for the median. C. The observed 2.5% (grey) and 97.5% (blue) percentiles are presented with dashed lines, and the 95% confidence intervals for the corresponding model predicted percentiles are shown as light dark grey and blue fields, respectively.

The pooled plasma concentration-time profiles of the anti-IL17 antibody by CRDS (triangles) and AMS (circles) spectroscopy in C57BL/6J mice are shown in Fig 4.

The ^14^C-labeled anti-IL17 plasma concentration-time profiles by CRDS (triangles) and AMS (circles) spectrometry after an IV dose of 2.1 nCi showed a biexponential decay. The observed profile means (lines) are superimposed in Fig 4A demonstrating the equivalence of these two ^14^C quantification methodologies. Eigenmann et al have recently reported on the ^125^I-labeling of the same model antibody (anti-IL17) and its pharmacokinetics and bio-distribution in mice after IV dosing of 66 nmol/kg anti-IL17 using a 600 nCi ^125^I-labeled anti-IL17 as microtracer [11]. For comparison, these plasma concentration-time profiles are also shown in Fig 4A, for which the experimental details where reported elsewhere [11]. Population PK modeling was applied to describe the pharmacokinetics data for anti-IL17. The final estimated parameters of the linear two-compartment model are listed in Table 1. The goodness-of-fit diagnostics indicated that the concentration profiles were described adequately well by the model and that the residuals were normally distributed. The high precision of the population parameter estimates further demonstrated that the pharmacokinetic data for the anti-IL17 antibody were well described indicating that the pharmacokinetic profiles are equivalent and are independent of the applied labeling or analytical technology. The derived volume of the central compartment (49.5 mL/kg, Table 1) was approximately equal to the plasma volume of mice (50 mL/kg with a body weight of 0.02 kg [18]) and the estimated systemic clearance of 6.7 mL day^-1^ kg^-1^ for the anti-IL17 (Table 1) was within the expected range of 3–16 mL day^-1^ kg^-1^ [27].The estimated effective half-life of 9.8 days for the anti-IL17 by the population approach was in agreement with the ones derived by non-compartmental analysis of 10.4, 10.0 and 11.1 days for the data derived by AMS, CRDS and γ-counting, respectively. The prediction corrected visual predictive check (PCVPC) for the molar plasma concentration profile of anti-IL17 are shown in Fig 4B and 4C. The PCVPC demonstrates graphically that the simulations from the 2-compartment linear model are able to reproduce well both the central trend and the variability in the observed data. The median (black line in Fig 4B) and the observed 2.5% and 97.5% percentiles (dotted lines in Fig 4C) of the prediction-corrected plasma concentrations are within the simulation-based 95% confidence intervals. This shows that the profiles with the biexponential behaviour for the anti-IL17 with a ^14^C nanotracer measured by either CRDS or AMS and for the anti-IL17 with a ^125^I- microtracer by γ-counting are in concordance.

**Table 1:** Pharmacokinetic parameter estimates for anti-IL17 monoclonal antibody in mice. CV. Coefficient of variation in percentage.NA. not applicable.

Parameter (Units)	Final Estimate	CV(%)
**PK parameters**		
V_c_ (mL kg^-1^)	49.5	6.05
CL (mL day^-1^kg^-1^)	6.69	3.68
Q (mL day^-1^kg^-1^)	84.3	8.05
V_p_ (mL kg^-1^)	44.7	8.42
**Inter-subject variability**		
ω^2^ (V_p_)	0.0871	32.6
**Residual variability**		
σ proportional	0.153	11.6
Derived Parameter (Units)	Final Estimate	CV(%)
t1/2_α_ (day)	0.190	NA
t1/2_β_ (day)	9.90	NA
effective t1/2 (day)	9.76	NA

Thus, these results demonstrate that CRDS and ^14^C-tracers can be used in antibody pharmacokinetic studies giving identical results to the traditional methodology. In 2015, Vlaming et al [28] showed for the first time that the linear pharmacokinetics of a ^14^C-labeled recombinant human protein could be successfully determined by application of microdosing combined with AMS early in the drug development process. For antibodies with non-linear pharmacokinetics due to target interaction, these novel techniques can be applied for studies were a ^14^C-tracer is added to a sub-pharmacological active dose so called intra-target microdosing. The ^14^C-tracer measurement would be an orthogonal analysis method to the traditional ELISA technique but without any interference by target and immune response. Together with model-based simulations this may allow quantitative extrapolation of the human exposure to the intended therapeutic dose [5].

### Bio-distribution

In addition to the concentration-time profiles in plasma, tissue concentrations with and without whole body perfusion were determined over time. The total tissue concentrations in kidney, liver, lung, muscle, skin and spleen were significantly lower than the plasma concentrations for anti-IL17 giving rise to mean antibody bio-distribution coefficients (ABC, tissue to plasma ratios in percentage) between 0.7 and 36%. The observed means of ABCs (CV%) are for kidney 11.1 (11.4), liver 6.57 (16.4), lung 14.3 (18.5), muscle 2.58 (12.3), skin 23.9 (31.9) and spleen 7.27 (21.3) based on total tissue concentrations without perfusion. The lowest ABC of 2.58 is found for muscle, followed by liver, spleen, kidney, lung and skin. The ABCs (CV%) are similar to the ones found for the ^125^I-labeled microtracer (no perfusion) by Eigenmann et al [11], e.g. for kidney 10.0 (14.2), liver 8.60 (8.04), lung 17.9 (14.7), muscle 3.76 (9.43), skin 11.2 (21.6) and spleen 6.12 (9.72). The found ABCs based on total tissue concentrations are in good agreement with the ABCs reported by Shah and Betts [29] for an IgG antibody. Comparing however ABCs between perfused and non-perfused animals, a significant difference was observed for well-perfused organs such as the kidney, liver, lung and spleen. The ABCs for perfused kidney and liver were < 10-fold lower, followed by spleen and lung with 2–3 fold lower ABCs as compared to non-perfused tissue. For muscle and skin, there was no difference in ABCs between perfused and non-perfused organs. The observed means of ABCs (CV%) were for kidney 1.20% (5.99) at day 1, 1.41% (16.6) at day 7 and 7.16% (19.5) at day 14, for liver 1.04% (69.9), lung 8.82% (45.4), muscle 2.23% (24.2), skin 28.2% (29.6) and spleen 2.53% (12.3) based on total tissue concentrations with perfusion. For kidney, the ABCs of anti-IL17 after perfusion increased over time, which was not seen for other tissues or without perfusion. Our general findings of lower ABCs in well-perfused organs after whole body perfusion were also observed for other antibodies [30, 31].

Recently, fractional residual plasma volumes in the different tissues of the mouse were determined by Boswell et al [17] and Eigenmann et al [11] using either ^99m^Tc labeling of red blood cells or ^125^I-labeled human serum albumin as plasma tracers. The fractional residual plasma volumes determined by Eigenmann et al [11] were 13.7% for lung, kidney 10.8%, liver 9.4%, spleen 6.5%, skin 1.7% and 0.9% for muscle, respectively. The low fractional residual plasma volumes of muscle and skin are in agreement with our findings of similar ABCs for muscle and skin with and without perfusion. In order to compare directly the bio-distribution results derived by perfusion in this study with their findings, the total tissue concentrations of anti-IL17 without perfusion were corrected by the residual plasma volumes and corrected ABCs were calculated.

**Fig 5:**
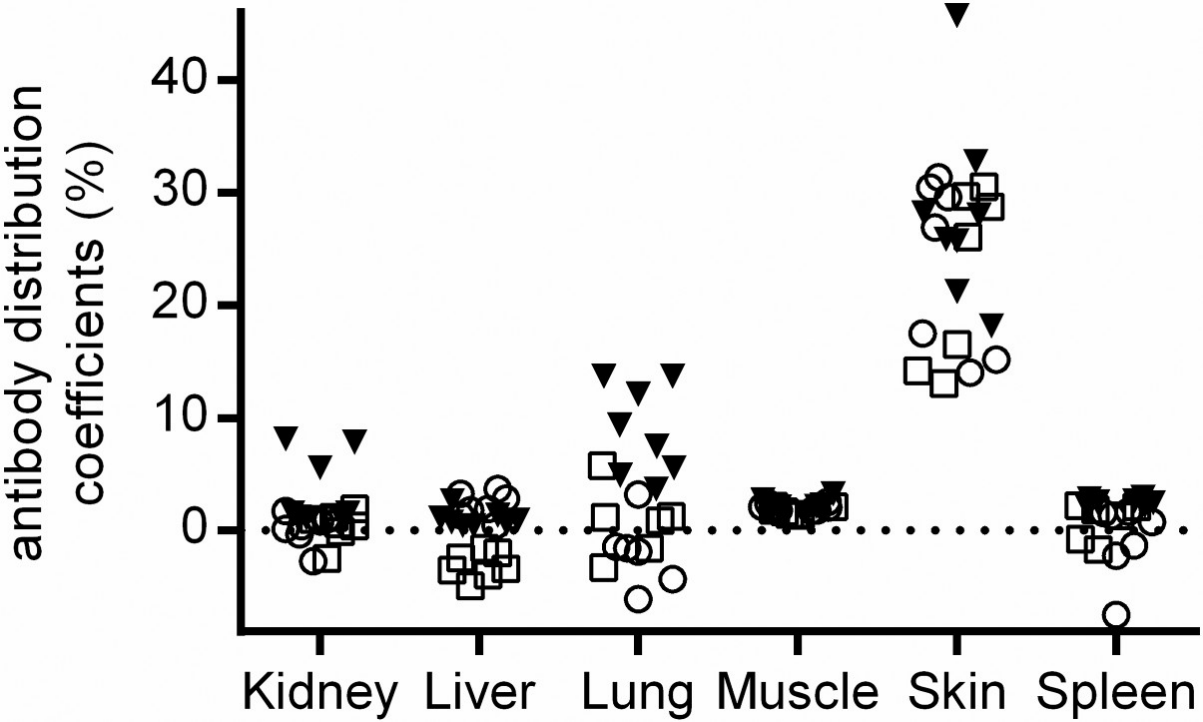
A comparison between antibody bio-distribution coefficients (ABCs) in mice by CRDS either based on perfused tissue concentrations or corrected tissue concentrations by residual plasma volumes. The filled triangles present perfusion based ABCs. The open squares and circles present calculated ABCs using either residual plasma volumes by Eigenmann et al [11] or Boswell et al [17], respectively. Dotted line presents zero line.

In Fig 5, a comparison is shown between the individual ABCs derived by whole body perfusion and the ones derived by corrected tissue concentrations based on residual plasma volumes. The ABCs derived by perfusion are given in filled triangles and the calculated ABCs corrected by residual plasma volumes in tissues are presented by open symbols (squares and circles, residual plasma volumes reported by Eigenmann et al [11] and Boswell et al [17], respectively). It can be seen that the ABCs derived after tissue perfusion and by correction with residual plasma volumes are in good agreement. The negligible tissue concentrations in liver and spleen after correction with the amount of drug in residual plasma is in line with significantly reduced ABCs to 1–3% of plasma concentrations after perfusion in our study. Due to discontinuous capillaries in the liver and spleen, no simple differentiation between extravascular and vascular antibody concentration can be made [11]. In the case of the kidney, however, the low ABC (< 2%) can be interpreted as negligible extravascular concentration of the antibody due to continuous capillary in the kidney. An increase of the ABC from 1.2% to 7% at day 14 was only observed with the perfusion technique suggesting an accumulation of the anti-IL17 in the kidney over time. For the lung, a higher mean ABC value of 8.8 was observed by perfusion compared to the calculated, corrected ABCs with an average value of around zero. Tissues with low residual plasma volumes (< 2%), such as muscle and skin, had similar ABCs across the different methodologies used, e.g. mean ABCs between 1.70 and 2.58 for muscle and 22.9 and 28.2% for skin, respectively. As the ABCs by residual plasma volume correction [11, 17] or by whole body perfusion are largely in agreement, the technique of the whole body or tissue perfusion coupled with CRDS spectrometry might be a direct and sensitive technique to determine extravascular tissue concentrations of an antibody in vivo more precisely.

## Conclusions

In this report, we demonstrate the use of a ^14^C-nanotracer and CRDS in a pharmacokinetic study of a therapeutic antibody. The ^14^C CRDS spectrometry gave equivalent pharmacokinetic results as compared to AMS, the correlation being particularly strong in plasma, a homogeneous matrix. In addition, the pharmacokinetic data of the ^14^C labeled nanotracer could be well described by a population PK model and compared well with the one of a ^125^I-labeled microtracer by traditional γ-counting. Thus, trace ^14^C-radiolabels and CRDS are therefore an ultrasensitive approach in antibody pharmacokinetics and bio-distribution studies and will be a key component to better understand pharmacokinetics and pharmacodynamics of new therapeutic antibodies especially in combination with positron emission tomography and pharmacodynamic read-outs for antibodies targeting tissues and cell populations. Novel experimental techniques, such as intra-targeting microdosing together with model-based approaches, may allow the addressing of non-linear pharmacokinetics of antibodies and the extrapolation of human exposure to the therapeutic dose in clinical studies [5]. In addition, CRDS is also suited for the quantification of absorption, metabolism and excretion of small molecule drugs in preclinical and both phase 0 and phase I microtracing/microdosing designs. The 2.1 nCi dose applied in this study for a 0.02 kg mouse would be equivalent to a 5.7 μCi dose being given to a 70 kg human. This value is significantly below the 50–100 μCi doses in standard ADME studies and above the 1 μCi (or less) sized dose typical of AMS human ADME [32]. However, a 100-fold dilution of the plasma samples in this study was needed to not exceed the dynamic range of AMS, but is still within the CRDS dynamic range. Thus, future studies with CRDS should be conducted with minimum of a few tens of pCi amounts for mouse and there will be no concerns for any dilutions with a detection limit in plasma at ~2 Modern, equivalent to 0.570 dpm/mL (above the baseline). In the near term, CRDS systems will complement AMS for biomedical applications in preclinical and clinical pharmacology and will be much less expensive to acquire and to operate than AMS. It is anticipated that as the CRDS technology matures it will find application in many bioanalytical fields, especially since advanced training in AMS is not required in order to operate the systems. Some examples of these include: (1) In vitro and in vivo models such as organs on a chip systems and in situ perfusion experiments where very small sample volumes should be drawn in order not to perturb the experimental setup yet very high measurement sensitivity should be attained (2) In vitro metabolism studies for highly stable molecules, where accurate quantitation of low abundance metabolites for which there are no available metabolite standards is required (3) Human and animal drug metabolism studies where either reduced radioactive dose or enhanced analytical sensitivity are desired (4) Microdose PK, bioavailability and DDI studies in man, especially in vulnerable populations. (5) Clinical diagnostics for personalized medicine. It is therefore hoped that with further validation of the CRDS technology applications, the systems will allow more human-relevant data to be generated earlier in the drug development process, speeding development decisions whilst also reducing the overall radioactivity exposure of clinical trial subjects and researchers.
